# Angiographic and Clinical Impact of Successful Manual Thrombus Aspiration in Diabetic Patients Undergoing Primary PCI

**DOI:** 10.1155/2014/263926

**Published:** 2014-04-02

**Authors:** Mohamed Shehata

**Affiliations:** Department of Cardiology, Faculty of Medicine, Ain Shams University Hospital, Abbasia Square, P.O. Box 11381, Cairo, Egypt

## Abstract

*Background*. Diabetes mellitus is associated with worse angiographic and clinical outcomes after percutaneous coronary intervention (PCI). *Aim*. To investigate the impact of manual thrombus aspiration on in-stent restenosis (ISR) and clinical outcome in patients treated by bare-metal stent (BMS) implantation for ST-segment elevation myocardial infarction (STEMI). *Methods*. 100 diabetic patients were prospectively enrolled. They were randomly assigned to undergo either standard primary PCI (group A, 50 patients) or PCI with thrombus aspiration using Export catheter (group B, 50 patients). The primary endpoint was the rate of eight-month ISR. The secondary endpoint included follow-up for major adverse cardiac events (MACE). *Results*. Mean age of the study cohort was 59.86 ± 8.3 years, with 64 (64%) being males. Baseline characteristics did not differ between both groups. Eight-month angiogram showed that group B patients had significantly less late lumen loss (0.17 ± 0.35 versus 0.60 ± 0.42 mm, *P* < 0.001), with lower incidence of ISR (4% versus 16.6%, *P* < 0.001). There was a trend towards lower rate of MACE in the same group of patients. *Conclusion*. In diabetic patients undergoing primary PCI, manual thrombus aspiration (compared with standard PCI) was associated with better ISR rate after BMS implantation.

## 1. Introduction

Acute myocardial infarction (MI) with ST-segment elevation is caused by rupture or erosion of an atherosclerotic plaque, initiating intraluminal thrombosis resulting in occlusion of a coronary artery [1–3]. Primary percutaneous coronary intervention (PCI) is the preferred treatment for MI with ST-segment elevation and is effective in opening the infarct-related artery [4–6]. However, microvascular obstruction with diminished myocardial perfusion occurs in a large proportion of patients with patent epicardial vessels after primary PCI, and this event is associated with an increased infarct size, reduced recovery of ventricular function, and increased mortality [7–11]. Microvascular obstruction is related to the embolization of plaque or thrombotic material downstream in the infarct-related artery [12, 13]. Embolization can occur spontaneously or by means of mechanical fragmentation during PCI [12–15]. The high frequency of suboptimal myocardial reperfusion after primary PCI has resulted in the development of various devices to protect the microcirculation [16–24]. Other proposed mechanisms contributing to microvascular obstruction and dysfunction include reperfusion injury, production of oxygen free radicals, neutrophil activation, endothelial and myocyte edema, loss of antioxidant enzymes, cardiomyocyte apoptosis, loss of endothelial mediated vasomotion, alteration of sympathetic innervation, plugging of platelets and neutrophils, epicardial coronary vasoconstriction, and increased myocardial cell calcium level [25]. It is proposed that the resulting cellular dysfunction, apoptosis, and necrosis mediate myocardial stunning, no-reflow, reperfusion arrhythmias, and additional loss of myocardium (lethal reperfusion injury) [26].

Diabetes mellitus is an important risk factor for poor outcome after PCI using bare-metal stents [27–29]. A more diffused and accelerated form of atherosclerosis in diabetic patients, accompanied by small vessel size, long lesions, and greater plaque burden, may contribute to the well-documented increased risk of restenosis after stent implantation in these patients [30]. In the current study, the author sought to explore the impact of upfront manual thrombus aspiration on angiographic (in-stent restenosis) and clinical outcomes, in diabetic patients treated by bare-metal stent implantation for acute ST-segment elevation myocardial infarction (STEMI).

## 2. Methods

### 2.1. Study Design and Data Collection

100 consecutive diabetic patients suffering from acute STEMI were prospectively enrolled in this study. They were referred to the catheterization laboratory on emergency basis, after being presented to the emergency department (ED), in the period between March 2011 and January 2013. All included patients suffered from insulin-dependent diabetes mellitus (IDDM). Other inclusion criteria included symptoms suggesting acute myocardial ischemia lasting >30 minutes, the onset of symptoms <12 hours before presentation to ED, and ST-segment elevation of >0.1 mV in two or more leads on the electrocardiogram (ECG). Exclusion criteria included patients undergoing rescue PCI after thrombolysis, patients with prior history of unstable angina or MI, those with prior PCI or coronary artery bypass graft (CABG) surgery, and those with congenital heart disease or any myocardial disease apart from ischemia. Patients with limited life expectancy due to coexistent disease, for example, malignancy, were excluded. After enrollment and before coronary angiography, patients were randomly assigned in 1 : 1 fashion to undergo either standard PCI (group A) or PCI with thrombus aspiration (group B), according to a computer-generated random series of numbers. Randomization was performed by block randomization (blocks of 10 patients). Physicians participating in PCI procedures were unaware of block randomization. Before inclusion, informed written consent was obtained from each patient and the study protocol was reviewed and approved by our local institutional human research committee, as it conforms to the ethical guidelines of the 1975 Declaration of Helsinki, as revised in 2008.

### 2.2. Definition of Risk Factors of Coronary Artery Disease

The presence of hypertension was defined as systolic blood pressure ≥140 mmHg and/or diastolic blood pressure ≥90 mmHg, previously recorded by repeated noninvasive office measurements, which led to life-style modification and/or intake of antihypertensive drug therapy [31]. The presence of diabetes mellitus was defined as fasting plasma glucose ≥126 mg/dL and/or two-hour postglucose load ≥200 mg/dL or specific antidiabetic drug therapy intake [32]. Dyslipidemia was defined as LDL cholesterol >100 mg/dL and/or serum triglycerides >150 mg/dL and/or HDL cholesterol <40 mg/dL (<50 mg/dL in women) [33].

### 2.3. PCI and Medications

Coronary angioplasty and stent implantation were performed according to institutional standards. For all patients, the first procedural step was the passing of a floppy, steerable guide wire through the target culprit lesion; direct stenting was left to operator's discretion and usually performed in patent vessel with no or mild calcification. In patients in the conventional PCI group (group A), this step was followed by balloon dilation to establish antegrade flow. Concerning patients in the thrombus aspiration group (group B), this step was followed by the advancing of the 6F Export Aspiration Catheter (Medtronic, Minneapolis, MN; crossing profile, 0.068 in.) into the target coronary segment during continuous aspiration. Aspiration was started proximal to the occluded site, gently pushing the catheter through the occlusion and then pulling it in a proximal direction, keeping negative pressure even when the occlusion was crossed or when there was no longer back bleeding in the syringe. Withdrawal of the catheter from the artery and from the guiding catheter was performed with permanent negative pressure. When necessary for stent delivery, balloon dilation was performed before stenting. In all patients, after the restoration of antegrade flow, intracoronary nitrates were given to ensure maximal epicardial vasodilatation, to determine the size and length of the required stent, and to facilitate stent placement. All placed stents were bare-metal stents. Pharmacological treatment before PCI included the administration of aspirin (a loading dose of 500 mg), heparin (70 IU/kg), and clopidogrel (a loading dose of 600 mg). All patients also received the glycoprotein IIb/IIIa inhibitor abciximab with an intravenous procedural bolus of 0.25 mg/kg followed by a continuous intravenous infusion of 0.125 *μ*g/kg/min for 12 hours and postprocedural infusion without heparin.

The primary endpoint was the rate of in-stent restenosis after eight months of follow-up, defined as angiographic luminal diameter stenosis by >50% using quantitative coronary angiography (QCA). The secondary endpoint included follow-up for major adverse cardiac events (MACE), that is, a composite of death due to cardiac cause, nonfatal myocardial infarction, and target lesion revascularization (TLR).

### 2.4. Quantitative Coronary Angiography (QCA)

Intracoronary nitroglycerine was administered before the initial, final, and follow-up angiograms to achieve maximal vasodilatation. Thrombolysis in myocardial infarction (TIMI) flow grade was recorded individually when flow restoration attempts were finalized. Contrast-filled guide catheters were used as the reference standard in QCA. Matched end-diastolic frames of the angiograms before and after PCI and at eight-month follow-up were analyzed using a contour detection minimum cost algorithm (QCA-CMS Version 3.0, MEDIS, Leiden, The Netherlands). Restenosis was defined as a stent stenosis >50% in diameter anywhere within the stent and/or within the 5 mm borders proximal or distal to the stent. Late lumen loss was defined as the difference between minimal lumen diameters (MLD) immediately after PCI and on eight-month follow-up angiography. QCA analyses were performed by an experienced technician who was blinded to patients' assignment.

### 2.5. Statistics

All continuous variables were statistically described in terms of mean ± standard deviation (±SD). Categorical variables were described with absolute and relative (percentage) frequencies. Comparison of continuous variables between the study groups was done using Student's *t*-test. For comparing categorical data, Pearson's Chi-square test was performed. *P* values were used to describe significance. All statistical calculations were done using Statistical Package for Social Sciences (SPSS for Windows) software (version 15.0, SPSS Inc., Chicago, IL, USA).

## 3. Results

### 3.1. Baseline Clinical Characteristics

A total of 100 consecutive IDDM patients suffering from acute STEMI were prospectively enrolled in the current study, which comprises 50 patients randomly assigned to undergo conventional PCI (group A) and 50 others randomly assigned to undergo PCI with thrombus aspiration (group B). The mean age of the whole study cohort was 59.86 ± 8.3 years, with 64 (64%) being male patients. The two groups were matched regarding age, gender, and risk factors of coronary artery disease (CAD). No significant difference was recorded between the two groups; concerning baseline mean serum HbA1c levels.  Table 1 shows baseline clinical characteristics of the two study groups. All patients underwent follow-up coronary angiography after eight months, except for six patients. Five (group A: three patients, group B: two patients) patients had definite late (>30 days after primary PCI) stent thrombosis and target lesion revascularization, while one patient belonging to group A died after 220 days, and thus a possible stent thrombosis is considered. So, follow-up angiograms were available in 94 (94%) patients (group A: *n* = 46, group B: *n* = 48). Blood samples were drawn for HbA1c quantitation at the day of follow-up of coronary angiography and revealed almost the same mean results (group A: 7.02 ± 0.3%, group B: 7.07 ± 0.4%; *P* > 0.05).

### 3.2. Lesion and Procedure Characteristics

Table 2 shows lesion and procedure characteristics. No statistically significant difference was recorded between the two study groups regarding lesion length, stent length, need for stent postdilatation, and frequency of stenting of each coronary artery. However, incidence of angiographic no-reflow was significantly less in group B patients (*P* < 0.05). Moreover, more patients in the same group showed TIMI III flow grade after culprit lesion stenting (*P* < 0.05). No serious periprocedural complications were recorded in both study groups.

### 3.3. Quantitative Angiographic Outcomes

Angiographic measures are shown in Table 3. Angiographic follow-up was obtained for 98 patients (98%), at a mean of 247.1 ± 27.4 days. Late luminal loss was found to be less in the group B (0.17 ± 0.35 mm versus 0.60 ± 0.42; *P* < 0.001). Also, the percentage of stenosis (in-stent restenosis) at follow-up was less in the same group (22.2 ± 9.7 in group B versus 35.37 ± 14.46 in group A; *P* < 0.001). The rate of in-stent restenosis (primary endpoint), defined as luminal diameter stenosis of >50 percent, was 4% (*n* = 2) in group B versus 16.6% (*n* = 8) in group A (*P* < 0.001).

### 3.4. Clinical Events

Clinical follow-up data revealed that 11 patients experienced major adverse cardiac events (MACE) as shown in Table 4, with no statistical difference between both study groups but a trend towards lower incidence in group B. Five patients suffered from definite stent thrombosis and TRL was done. Only one patient (belonging to group A) died during the follow-up period with possible stent thrombosis.

## 4. Discussion

The clinical importance of embolization of atherothrombotic materials from unstable plaques in patients with myocardial infarction with ST-segment elevation has been recognized [12, 15], and the use of thrombectomy aspiration devices to reduce distal embolization, preserving tissue-level perfusion, has been tested in several studies with conflicting results [16, 17, 20–24, 34–38]. Results achieved using different techniques to explore different aspects of microvascular integrity further enhance the strength of the tested hypothesis that distal embolization during primary PCI plays a significant role in the pathogenesis of microvascular obstruction. This was because thrombus aspiration significantly reduces the severity and extent of the phenomenon [39]. Despite early invasive strategies and optimal medical treatment, morbidity and mortality rates for STEMI continue to be high. Thus, further improvement of the initial therapy is necessary to improve patient outcome. The pivotal TAPAS (Thrombus Aspiration during Percutaneous coronary intervention in Acute myocardial infarction Study) trial of patients with STEMI, which compared a strategy with thrombectomy with a strategy without thrombectomy, showed a reduction in cardiac mortality at one year [38]. By contrast, other randomized, controlled studies did not observe superiority of thrombectomy over standard PCI with respect to surrogate endpoints of reperfusion success [40, 41]. However, meta-analyses in patients with STEMI showed a mortality-related benefit after thrombectomy compared with PCI alone [42, 43]. Therefore, the current guidelines of the European Society of Cardiology (ESC) on revascularization therapy in patients with STEMI reperfused by primary PCI have increased the recommendation class for manual thrombectomy from the previous class IIb, level of evidence B, to class IIa, level of evidence A [44]. In line with the ESC guidelines, thrombectomy is strongly recommended by the American Heart Association/American College of Cardiology (AHA/ACC) guidelines (class IIa, level of evidence B) [45]. The current study sought to evaluate the clinical and angiographic outcome of thrombus aspiration during primary PCI in diabetic patients presented with STEMI. Eligible patients were randomly assigned to either primary PCI with thrombus aspiration or standard PCI. Bare-metal stents were used in all patients. Bare-metal stents are still the only available option (especially on emergency basis) for percutaneous revascularization in most of centers located in developing countries. The current study was accomplished in one of these countries, where there is still a considerable cost burden concerning the use of drug-eluting stents (DESs). Patients who underwent primary PCI with thrombus aspiration showed significantly higher incidence of final TIMI III flow and less incidence of angiographic no-reflow phenomenon. Eight-month follow-up coronary angiography showed favorable QCA results concerning the same group of patients with statistically significant less incidence of in-stent restenosis, less percentage of diameter stenosis, and less late lumen loss. It is worth mentioning that mean culprit lesion length and mean used stent length did not differ between patients belonging to thrombus aspiration strategy and others belonging to standard PCI strategy. No recorded significant difference between both study groups concerning follow-up for MACE after the same period of time, that is, eight months. However, there was a recorded case of cardiac mortality, possibly stent thrombosis, and a case of definite stent thrombosis mounting to target lesion revascularization in the standard PCI group of patients. The presented results highlighted the favorable impact of thrombus aspiration, during primary PCI in diabetic patients, on incidence of in-stent restenosis and consequently the other QCA parameters. Although the favorable clinical impact of thrombus aspiration did not reach a statistical significance (when compared to the angiographic impact), numerical values still show that adopting thrombus aspiration strategy was associated with less incidence of MACE. A larger number of patients for a longer follow-up period might magnify the obtained follow-up clinical results. The author hypothesized that adopting thrombus aspiration strategy in this clinical setting could improve both angiographic and clinical outcomes encountered in diabetic patients, especially those who are managed using bare-metal stents.

### 4.1. Comparison with Other Studies

Previous studies demonstrated safety and merits of using thrombus aspiration devices in primary PCI prior to coronary stenting, especially concerning myocardial salvage and improved myocardial reperfusion [20, 22, 46–49]. However, this was not always associated with reduction of infarct size [42, 47]. The current study results showed better early angiographic outcomes upon using thrombus aspiration device in diabetic patients in the setting of primary PCI, in the form of higher frequency of TIMI III flow and less frequent no-reflow phenomenon. This was also demonstrated by previous trials [41, 49, 50], not specifically targeting diabetic patients. A previous trial reported the favorable impact of using thrombus aspiration (in primary PCI) on incidence of in-stent restenosis [51]. However, this trial also was not addressing diabetic patients in particular. To the best of the author's knowledge, the current study is a unique one, exploring the impact of aspiration thrombectomy on in-stent restenosis in diabetic patients. The current study utilized export aspiration catheter for upfront thrombus aspiration as was previously proven to be effective and safe in primary PCI [41, 49, 51]. In agreement with the results of the current study, prior trials reported satisfactory intermediate and long-term follow-up clinical outcomes, favoring adopting thrombectomy strategy in primary PCI settings [49, 51]. It is worth mentioning that all patients in both current study groups were assumed to exhibit more or less controlled blood glucose during the follow-up period as shown in mean HbA1c levels tested shortly before follow-up angiograms. So, quality of glycemic control during the follow-up period was not a determining factor for incidence of in-stent restenosis in the present study. A prior study stated that proper glycemic control was associated with lower incidence of in-stent restenosis at six-month follow-up, after acute STEMI [52]. However, thrombectomy aspiration procedure was not included in the study protocol.

### 4.2. Clinical Implications

Diabetes mellitus was proven to be associated with worse angiographic (in-stent restenosis, target lesion revascularization, and stent thrombosis) and clinical (MACE) outcomes after PCI, regardless of the type of the used stent [53]. That is why interventionalists are always attempting to optimize the procedural circumstances, in order to achieve satisfactory short- and long-term results in diabetic patients. The current study highlighted the favorable impact of thrombus aspiration on late angiographic and clinical outcomes in diabetic patients undergoing primary PCI, using bare-metal stents. The author recommends adopting thrombus aspiration strategy, whenever possible, in the aforementioned clinical setting.

### 4.3. Limitations of the Study

The data presented in our study only apply for patients defined by inclusion and exclusion criteria. Moreover, this is a single-centre study with a relatively small sample size of the cohort. 2D-QCA was used for angiographic assessment in the present study. Subsequent studies are needed to verify the obtained results using 3D-QCA and/or intravascular ultrasound (IVUS). Follow-up period in the current study was limited to eight months. A longer follow-up period is warranted for confirmation of the current study results, perhaps with the use of DESs. Assessment of successful tissue reperfusion and infarct size was not included in the current study, as these parameters were outside the scope of the study protocol.

## 5. Conclusion

Successful upfront manual thrombus aspiration (in diabetic patients on insulin therapy) during primary PCI showed beneficial effects on the reduction of in-stent restenosis after bare-metal stent implantation compared with standard PCI.

## Figures and Tables

**Table 1:** Baseline characteristics of the two study groups.

Item	Group A (*n* = 50)	Group B (*n* = 50)	*P* value*
Age (years)	59.4 ± 7.4	60.32 ± 9.2	>0.05
Males	33 (66)	31 (62)	>0.05
Hypertension	32 (64)	30 (60)	>0.05
Smoking	9 (18)	12 (24)	>0.05
Dyslipidemia	18 (36)	16 (32)	>0.05
Family history of CAD	8 (16)	6 (12)	>0.05
HbA1c (%)	7.1 ± 1.3	7.2 ± 1.6	>0.05
Serum creatinine (mg%)	1.1 ± 0.3	1.0 ± 0.4	>0.05
Door to balloon time (minutes)	73.7 ± 39.2	77.7 ± 36.4	>0.05
Killip class ≥3	4 (8)	6 (12)	>0.05

CAD: coronary artery disease.

Age, HbA1c, serum creatinine, and door to balloon time are presented as mean ± standard deviation. Other variables are presented as number (percentage).

^∗^ *t*-test and Pearson's Chi-square test.

**Table 2:** Procedural characteristics of the two study groups.

Item	Group A (*n* = 50)	Group B (*n* = 50)	*P* value*
Lesion length (mm)	14.4 ± 3.2	13.5 ± 4.2	>0.05
Stent length (mm)	16.6 ± 5.3	16.7 ± 5.8	>0.05
Stent diameter	2.77 ± 0.5	2.8 ± 0.5	>0.05
Stent postdilatation	21 (42)	18 (36)	>0.05
TIMI 3 flow (after PCI)	20 (40)	38 (76)	**<0.05**
Angiographic no-reflow	10 (20)	2 (4)	**<0.05**
Infarct-related artery			
LAD	25 (50)	29 (58)	>0.05
LCx	10 (20)	8 (16)	>0.05
RCA	15 (30)	13 (26)	>0.05

TIMI: thrombolysis in myocardial infarction; LAD: left anterior descending; LCx: left circumflex; RCA: right coronary artery.

Lesion length and stent length are presented as mean ± standard deviation. Other variables are presented as number (percentage).

*t-test and Pearson's Chi-square test.

**Table 3:** Quantitative coronary angiography results: baseline and 8-month follow-up.

Item	Group A	Group B	*P* value*
After primary PCI^†^			
Reference vessel diameter (mm)	2.85 ± 0.60	3.11 ± 0.55	>0.05
Minimal lumen diameter (mm)	2.55 ± 0.40	2.73 ± 0.69	>0.05
Diameter stenosis (residual in-stent stenosis) (%)	9.5 ± 0.3	10.2 ± 0.54	>0.05
After follow-up coronary angiography^‡^ (8 months)			
Reference vessel diameter (mm)	2.82 ± 0.58	2.95 ± 0.35	>0.05
Minimal lumen diameter (mm)	1.95 ± 0.55	2.56 ± 0.33	**<0.001**
Diameter stenosis (ISR) (%)	35.37 ± 14.46	22.2 ± 9.7	**<0.001**
Late lumen loss (mm)	0.60 ± 0.42	0.17 ± 0.35	**<0.001**

PCI: percutaneous coronary intervention; ISR: in-stent restenosis.

All variables are presented as mean ± standard deviation.

*t-test; ^†^100 patients (group A: 50, group B: 50); ^‡^94 patients (group A: 46, group B: 48).

**Table 4:** Clinical events at 8-month follow-up.

Item	Group A (*n* = 50)	Group B (*n* = 50)	*P* value*
Cardiac death	1	0	
Stent thrombosis			
Definite	3	2	
Probable	0	0	
Possible	1	0	
Myocardial infarction			
STEMI	3	2	
NSTEMI	3	2	
Target lesion revascularization	3	2	
MACE	7	4	>0.05

STEMI: ST-segment elevation myocardial infarction; NSTEMI: non-ST-segment elevation myocardial infarction; MACE: major adverse cardiac events.

*Pearson's Chi-square test.
